# A review of complex hormone regulation in thyroid cancer: novel insights beyond the hypothalamus–pituitary–thyroid axis

**DOI:** 10.3389/fendo.2024.1419913

**Published:** 2024-07-22

**Authors:** Liu-han Chen, Tao Xie, Qian Lei, Yan-rui Gu, Chuan-zheng Sun

**Affiliations:** Department of Head and Neck Surgery section II, The Third Affiliated Hospital of Kunming Medical University, Kunming, Yunnan, China

**Keywords:** thyroid cancer, hormone, hormone system, growth hormone, insulin-like growth factor, estrogen, androgen, glucocorticoid

## Abstract

Like the ovaries and prostate, the thyroid exhibits characteristic hormone secretion and regulation. Thyroid cancer (TC), especially differentiated thyroid carcinoma, has typical sex-specific and age-specific hormone-driven clinical features. Previous research has primarily focused on the effects of thyroid stimulating hormone, thyroid hormones, and estrogens on the onset and progression of TC, while the roles of growth hormone (GH), androgens, and glucocorticoids have largely been overlooked. Similarly, few studies have investigated the interactions between hormones and hormone systems. In fact, numerous studies of patients with acromegaly have shown that serum levels of GH and insulin-like growth factor-1 (IGF-1) may be associated with the onset and progression of TC, although the influences of age, sex, and other risk factors, such as obesity and stress, remain unclear. Sex hormones, the GH/IGF axis, and glucocorticoids are likely involved in the onset and progression of TC by regulating the tumor microenvironment and metabolism. The aim of this review was to clarify the roles of hormones and hormone systems in TC, especially papillary thyroid carcinoma, as references for further investigations.

## Introduction

Precise production of hormones and regulation of hormone systems are essential for homeostasis and physical functions. Hormone dysregulation contributes to the onset and progression of many pathologies, including cancers. The hypothalamus plays a central role in regulation of both pituitary hormones and important hormone systems, especially the hypothalamic-pituitary-adrenal (HPA), hypothalamic-pituitary-gonadal (HPG), and hypothalamic-pituitary-thyroidal (HPT) axes, in various physiological and pathological processes.

Sex steroids control gonadal development, metabolism, and immunity. The HPG axis regulates secretion of sex hormones via activation of gonadotropin-releasing hormone, which is followed by release of follicle-stimulating hormone and luteinizing hormone from the anterior pituitary. Secretion of sex hormones is dynamic and influenced by various factors, such as emotional changes, stress, diet, and obesity.

Growth hormone (GH) secreted by the pituitary regulates development and metabolism of carbohydrates, proteins, and fats. Serum GH levels peak during early childhood and puberty, then gradually decrease with age. Stressors, such as low blood sugar and intense exercise, stimulate the release of GH (1). Moreover, GH indirectly stimulates production of insulin-like growth factor-1 (IGF-1) in the liver and kidneys, which regulates DNA production and cell division. The interaction between GH and IGF-1 may underlie physiological and pathological cell growth and proliferation.

The HPA axis regulates stress responses, energy balance, and immune function. In response to physical or psychological stress, corticotropin-releasing hormone (CRH) produced by the hypothalamus binds to receptors on the adrenal cortex to control the release of glucocorticoids. The HPA axis is regulated by negative feedback inhibition from glucocorticoids as well as genomic and non-genomic factors. Glucocorticoids are necessary for maintenance of normal physiological functions and processes, including metabolism, immune responses, mood, cognitive functions, reproduction, and development (2).

Thyroid cancer (TC) is the most common cancer of the endocrine system (3). The prevalence of TC is influenced by age, sex, inheritance, and radiation exposure. While most TC patients have normal levels of thyroid hormones (THs) and thyroid stimulating hormone (TSH), many studies have confirmed an association between changes in thyroid function and the onset and development of TC. Notably, TSH promotes growth of thyroid follicular cells, which potentially impact the onset and progression of TC. Similarly, both TSH and THs are associated with certain invasive clinicopathological characteristics and postoperative recurrence of TC (4). TH replacement therapy is the primary approach for long-term management of TC. After total thyroidectomy or lobectomy, TH replacement therapy is initiated to restore euthyroidism and serum TSH levels. However, aggressive TSH-suppressive therapy has limited or no benefits for many patients with differentiated thyroid carcinoma (DTC) (5). Therefore, hormonal regulation of TC is not limited to TSH and THs.

Many malignant tumors exhibit sex-specific differences in occurrence, malignancy, aggressiveness, and prognosis. Unlike endocrine organs, such as the mammary glands, prostate, ovaries, and testes, the thyroid plays a crucial physiological role in both males and females. Although changes to THs and TSH levels in patients with TC are very subtle, variations in levels of TSH, triiodothyronine (T3), and thyroxine (T4) have different effects on the occurrence and development of TC between males and females (6). Although the incidence is higher among females, TC tends to be more aggressive in males (7). TC in males typically presents with a higher propensity for extrathyroidal invasion, lymph node metastasis, and even distant metastasis. A retrospective clinical study reported that TC was more invasiveness in children and adolescents (8). Notably, more than half of adolescent males with TC have lymph node metastasis, while the number of adults and the elderly with TC and lymph node metastasis has decreased. In addition, acromegaly, which is characterized by high GH and IGF-1 levels, and has been associated with greater risks for thyroid diseases, including TC (9). Moreover, the association between stress, obesity, abnormal blood pressure and TC has been increasingly recognized (10). Therefore, the aim of this review is to clarify the functions of hormone and hormone systems in TC and the influences of age, sex, and other risk factors.

## Regulation of thyroid development and hormone secretion

The thyroid is the largest endocrine gland in the human endocrine system and indispensable for cellular differentiation and growth. The HPT axis serves as the main regulator of thyroid growth and the production and release of THs. The physiological functions of TSH, especially iodide uptake and production of THs, are activated by binding to the thyrotropin receptor (TSHR) (11). Additionally, various other factors, such as IGF-1, transforming Growth Factor β,GH, and prolactin, are involved in development and growth of the thyroid (12–14).

Exposure to chlordecone, a endocrine-disrupting chemical that mimics female hormones, has been associated with increased levels of TSH and sex steroid hormones *in utero (* 15). With sufficient iodine levels, the thyroid regulates the “growth spurt” that occurs at the onset of puberty of euthyroid children and adolescents, but seems to be regulated by significant increases in circulating sex steroids rather than TSH (16). Meanwhile, variations in sex steroid levels were reported to impact thyroid growth and TSH regulation in rats (17).

GH replacement therapy has been shown to increase serum T3 levels and the total volume of the thyroid gland in GH-deficient adults (18). IGF-1 is a significant modulator of the TSH response in adult thyroid cell differentiation and has an additive effect with TSH (19). Cheung et al. (20) found that IGF-1 promoted thyroid cell proliferation by potentiating the mitogenic activities of TSH. Goretzki et al. (21) demonstrated that TSH enhanced production of thyroid-specific autocrine IGF-1, which is crucial to growth of thyrocytes. Another study by Goretzki et al. (22) supported these findings and showed that the effects of TSH are concentration dependent in tumor cells. For example, TSH at 100 mIU/ml promoted growth of tumor cells, but had an inhibitory effect at higher concentrations. This study also found that TSH regulation of thyrocyte growth is controlled by locally produced IGF-1 (22). These findings emphasize the regulatory role of IGF-1 in the growth of human thyrocytes. Moreover, T3 is also necessary for secretion of GH secretion. However, IGF-1 is reported to suppress GH gene expression through a short negative feedback loop involving T3. Moreover, circulating levels of IGF-1 and TSH receptors have been associated with Graves’ disease (23).

**Figure 1:**
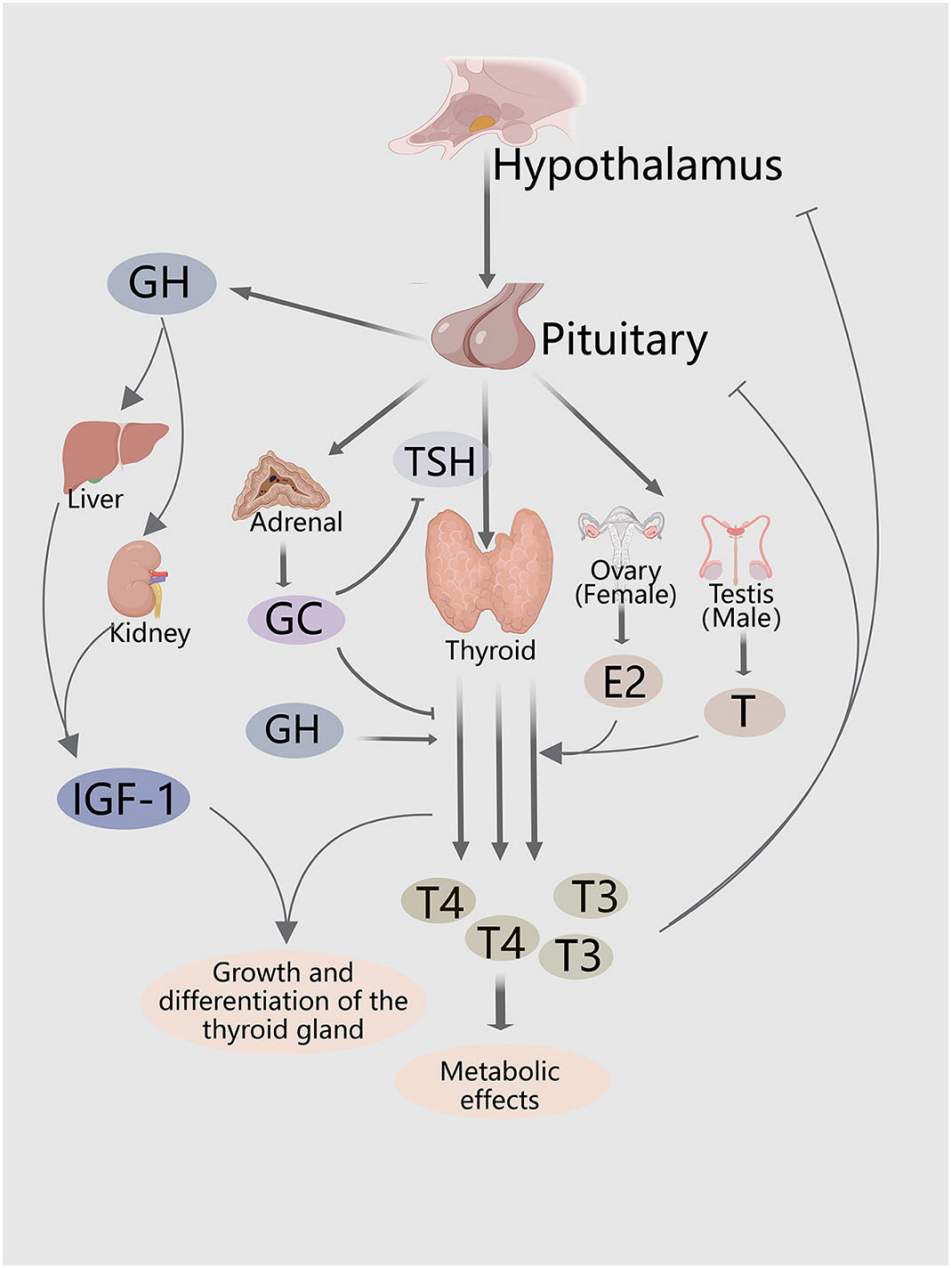
Hormones and hormone systems both directly and indirectly regulate thyroid growth, and thyroid hormone secretion. The HPT axis regulates thyroid proliferation and production of THs. The GH/IGF-1 axis and sex steroids indirectly promote secretion of THs, and thyroid growth. HPA axis inhibits secretion of THs and TSH.

In addition, cortisol levels influence secretion of THs. Cai et al. (24) demonstrated that serum levels of TSH and free T4 were significantly decreased in cortisol-producing adenomas (CPAs) as compared to healthy controls and asymptomatic adrenal incidentalomas. This study also revealed a negative association of cortisol, TSH, and T4 serum levels with CPAs. Furthermore, adrenalectomy was shown to reverse decreased serum levels of both TSH and free T4. However, GH and prolactin levels remained unchanged from baseline levels. These findings suggest that GH, IGF-1, sex hormones, and cortisol control thyroid development and secretion of THs (**Figure 1**).

### HPG axis and sex hormones

The incidence of TC is 3–5-fold greater in females than males (7, 25, 26). Endogenous estrogens play complex and ambiguous roles in the development of DTC, especially among females during perimenopause (27). Furthermore, higher levels of testosterone and androstenedione increase the risk of DTC in pre/perimenopausal women, but not men or postmenopausal women (28), suggesting that regulation of sex hormones in TC is complex and dynamic.

#### Estrogen, progesterone, and receptors

Estrogen is an important steroid hormone that regulates development and immune function. Polymorphisms of the estrogen receptor (ER) influence the responses of tissue to estrogens and contribute to oncogenesis (29). The involvement of the ER and progesterone receptor (PR) in TC has been established. For instance, a study by Vannucchi et al. (30) found that more than two-thirds of 182 patients with papillary thyroid cancer (PTC) were positive for estrogen receptor alpha (ERα) and PR. Meanwhile, Eldien et al. (31) reported that upregulated expression of ER and PR, in addition to advanced age, were significantly associated with primary TC. Additionally, pregnancy and delivery can influence the persistence and recurrence of TC (32).

The precise functions of female hormones in TC vary across different studies. A nationwide cohort study conducted in Korea (33) found that estrogen and related hormone receptors only slightly promoted progression of TC, while the absence of estrogen did not protect against disease onset. Although the risk of TC is increased after hysterectomy and oophorectomy, there is reportedly no significant correlation between the use of oral contraceptives and disease incidence (34). Moreover, studies on the effects of phytoestrogens on the development of TC have reported mixed results. For example, high intake of coumestrol was correlated to an increased risk of TC, while moderate intake of genistein provided some protection against thyroid macrocarcinomas in females (35).

Estrogen regulates the progression of TC through both classical genomic and non-genomic pathways (26). In the genomic pathway, estrogen enters the cell and forms a complex with ERα and estrogen receptor Beta (ERβ), which binds to estrogen-responsive elements to promote transcription of target genes (36). Additionally, membrane-associated estrogen receptors are involved in non-genomic signaling of estradiol (E2), which stimulates activation of various pathways associated with regulation of the cell cycle, signal transmission from membrane-based receptors, and immune and inflammatory responses (37). In PTC, these pathways are activated either by chromosomal rearrangement of the receptor tyrosine kinase (RTK),or the BRAF^V600E^ mutation (37).

In TC, estrogen and hypoxia promote generation of reactive oxygen species (ROS) through various mechanisms (38). Estrogen/ERα-dependent autophagy has been associated with production of ROS, activation of extracellular signal-regulated kinases 1 and 2 (ERK1/2), and the survival and growth of PTC cells (39). Excessive accumulation of ROS or the lack of proper detoxification mechanisms can be detrimental to cells and contribute to the occurrence of TC. Oxidative damage, particularly to DNA, can promote malignant transformation of thyroid tissues, while 2,4-dienoyl CoA reductase 1 (NADPH) oxidase generates substantial amounts of ROS, thus potentially enhancing the incidence of spontaneous mutations. Faria et al. (40) demonstrated that estrogen via NADPH oxidase 4 stimulates production of ROS, which can penetrate the nucleus, likely contributing to thyroid carcinogenesis, and that the thyroid glands of adult female, as compared to male, rats produce relatively higher amounts of hydrogen peroxide and relatively lower levels of antioxidant enzymes. Furthermore, estrogen and hypoxia have been reported to regulate hypoxia-inducible factor-1 and influence key molecular, cellular, and metabolic processes involved in progression of TC (38).

ER mediates production of ROS and promotes cellular proliferation (41, 42). The expression patterns of ERα and ERβ in TC are complex and multifaceted. PTC tissues have elevated expression of ERα as compared to adjacent non-tumor tissues (39). Kim et al. (43) suggested that ERα may play a role in the association between breast cancer and TC, although ERβ has been shown to act as an oncosuppressor. For instance, Magri et al. (44) reported that ERβ (−), as compared to ERβ (+), tumors were more likely to have vascular invasion. However, Dong et al. (45) found a positive correlation between ERβ2 and Ki-67 in female TC patients during perimenopause. Nevertheless, ERβ2 expression was lower in females of reproductive age with lymph node metastasis of PTC than without and positively associated with vascular endothelial growth factor expression in males aged 18–45 years, but not tumor size, extrathyroidal extension, or metastasis stage (45). Interestingly, undifferentiated thyroid stem and progenitor cells exhibited lower levels of ERβ as compared to differentiated human thyrocytes (46). Thus, low levels of ER expression may indicate dedifferentiation in TC (26).

ERs are expressed by various immune cells in the tumor microenvironment (TME) of TC and play various roles in tumorigenesis and inflammation (41, 47). As important constituents of the TME, immune cells contribute to tumor growth by facilitating metastasis of malignant cells. The BRAF^V600E^ mutation plays an important role in the estrogen responsiveness of TC by regulating ER expression (48). Additionally, a detailed comparison of glycosylation patterns of TC patients and healthy controls provided insights into abnormal changes to glycosylation of the Fc fragment of immunoglobulin (Ig) G1. Interestingly, among females, there were distinct changes in the incidences of most glycosylated forms starting at puberty or menopause that were associated with sex hormones and IgG glycans, with a particularly notable impact of E2 (49).

#### Androgens and receptors

Xu et al. (50) reported associations between serum levels of sex hormones and the pathological characteristics of PTC in males, as high serum levels of estrogens promoted proliferation of cancer cells, while androgens exhibited protective effects, as least to some extent. This finding is consistent with the clinical phenomenon of the higher incidence of PTC in females. However, the protective roles of androgens against the onset and progression of TC remain controversial (**Table 1**). Thus, the functions of androgens and androgen receptors (ARs) in the development of TC are complex and multifaceted.

**Table 1:** Studies that report the role of androgen/receptors in TC in addition to the dual effects of risk and protective factors.

Study	TC type	Androgen/receptors are risk factor or protective factor	Effects of androgen/receptors
Zhang et al (25)	FTC	risk factor	Inhibit tumor immunity
Jiang et al (51)	PTC	risk factor	Promote the migration, invasion and EMT process
Banu et al (52)	FTC and PTC	risk factor	Promote proliferation
Gupta et al (53)	ATC and PTC	protective factor	Induce senescence
O’Connell et al (54)	PTC and ATC	protective factor	Reduce PD-L1 promoter activation
Jones et al (55)	ATC	protective factor	Induce a G1 arrest
Chou et al (56)	PTC	protective factor	Decrease cell migration and the EMT process

Testosterone is an androgen that plays various physiological roles in the onset and progression of PTC (51). Banu et al. (52) suggested that testosterone, similar to E2, promotes growth and metastasis of PTC cells, although the underlying mechanisms remain unclear. Zhang et al. (25) showed that testosterone regulates progression of TC by reducing expression of tumor suppressor genes and tumor immunity. In addition, Jiang et al. (51) found that testosterone facilitated growth, invasion, and migration of PTC cells and epithelial-mesenchymal transition (EMT). Upregulation of Tnnt1 activates the P38/JNK pathway and promotes malignant behavior in PTC (51). Furthermore, Spirina et al. (57) revealed upregulated expression of AR in PTC, while Magri et al. (44) suggested that AR expression was associated with higher frequencies of capsular invasion. Similarly, Gupta et al. (53) observed increased migration, rather than invasion, by TC cells treated with 5α-dihydrotestosterone, demonstrating that androgens may contribute to the progression of TC.

However, many investigations found that androgens protective against TC (55). High expression of programmed death-ligand 1 (PD-L1) has been associated with aggressive forms of TC (58). O’Connell et al. (54) showed that AR activation decreased PD-L1 expression in TC cells via inhibition of the nuclear factor kB (NF-kB) signaling. Moreover, Gupta et al. (53) provided evidence that activation of AR promotes senescence and apoptosis of malignant TC cells. Similarly, Chou et al. (56) reported that overexpression of AR decreased cell migration and repressed epithelial-mesenchymal transition (EMT) in TC, indicating that the androgen-AR axis may protect males against TC, at least to some extent. The contradictory effects of sex hormones in TC may be explained by the different responsiveness to hormones and the influences of other hormones.

### HPA axis and glucocorticoids

For the past six decades, there has been a predominant focus on the immunosuppressive properties of glucocorticoids. However, recent studies have increasingly demonstrated that glucocorticoids also enhance inflammation and immunity (59). Nonetheless, current evidence is insufficient to support the involvement of glucocorticoids in the formation of the TME in TC. Upon binding to an appropriate glucocorticoid receptor (GR), glucocorticoids are involved in various physiological processes, including cell differentiation, metabolism, and proliferation (60). Crucially, glucocorticoids play significant roles in responses to stressors by facilitating energy production, inflammation suppression, and blood pressure regulation. Afrashteh et al. (10) demonstrated that stress and short-temperedness were directly related to the occurrence of TC, while sufficient sleep quantity and good sleep quality appeared to decrease this risk. The HPA axis and glucocorticoids, which serve as the primary regulators of stress, are potential predictors of the risk of TC in individuals experiencing high levels of stress. Lee et al. (61) found higher GR expression in males, as compared to females, with TC. Furthermore, GR expression was significantly higher in TC patients aged >45 years, suggesting that the pathobiological role of GR in TC might be associated with changes to the circadian rhythm of thyroid tumors (61). Additionally, Choi et al. (62) found that urine corticoid levels were slightly higher in male PTC patients.

Invitti et al. (63) reported that the prevalence of nodular thyroid disease was significantly higher in patients with Cushing’s disease. Although the direct cause of thyroid changes in relation to glucocorticoid excess remains unclear, other factors could potentially be involved, such as increased activity of corticotrophic cells or the presence of a growth factor that stimulates both corticotroph and thyrocyte proliferation. In addition, Zhang et al. (64) suggested a possible relationship between GR expression and thyroid adenomas, although this association was not established in TC. Conversely, Melnik et al. (65) reported that the synthetic glucocorticoid dexamethasone suppressed metastasis of follicular thyroid cancer (FTC) cells, but had no effect on benign and recurrent FTC. Relatively few studies have explored potential between the HPA axis and the onset and progression of TC. Although glucocorticoids have different effects on the regulation of stress, immunity, and inflammatory responses in the hormone environment depending on sex and age, these reactions may directly or indirectly participate in the onset and development of TC. Therefore, a thorough evaluation involving a larger patient cohort is needed to clarify the roles of glucocorticoids and GRs in TC.

## GH and GH/IGF axis

Studies investigating the mechanisms of GH in TC have established that growth hormone-releasing hormone (GHRH), a peptide hormone secreted by the hypothalamus that regulates GH synthesis and secretion in the pituitary, plays a role in TC. GHRH expression and GHRH receptor mRNA have been identified in thyroid cells, and inhibition of GHRH has been found to suppress growth and promote apoptosis of TC cells (66). However, the specific functions of GH in these processes remain unclear. Studies exploring the roles of the GH/IGF-1 axis in TC have primarily focused on individuals with acromegaly, who typically exhibit elevated GH and IGF-1 levels. Nonetheless, the results of these studies have yielded conflicting findings. Most research in this area has predominantly concentrated on three main aspects: (i) whether individuals with acromegaly are at a greater risk of TC; (ii) whether the onset and progression of TC in acromegaly patients are associated with GH/IGF-1 levels; and (iii) whether there are any sex differences of TC among patients with acromegaly. However, further investigations are still required to precisely address these questions.

### Relationship between GH/IGF excess and TC

Colorectal cancer and thyroid carcinoma, especially PTC, are the most frequently occurring malignant tumors among individuals with acromegaly (67). Most studies (68–71) report an increased risk of TC in acromegaly patients. More aggressive tumor behavior of TC has also been associated with acromegaly (72). However, other studies (73–75) found no significant difference in TC occurrence between acromegaly patients and the control group, despite a higher incidence of thyroid diseases (**Table 2**).

**Table 2:** Recent single and multicenter retrospective cohort and case-control studies that report the prevalence of TC in acromegaly (The last three years of research in PubMed).

Study	Year	Number of patients in total	Patients with cancer (n/% in total cohort)	Patients with TC (n/% in cancer cohort)
Durmuş et al (68)	2022	179	24 (13.4%)	11(45.8%)
Xiao et al (69)	2023	1738	67(3.9%)	33(49.3%)
Oguz et al (70)	2023	394	63(16.0%)	26(41.3%)
Esposito et al (75)	2021	1296	186(14.4%)	3(1.6%)
Plotuna et al (71)	2023	34	5(14.7%)	3(60%)

The pathogenesis of TC varies among acromegaly patients. Some studies (9, 76) suggested a potential link between the activation of IGF-1 and the development of TC. Several case series reported that somatostatin receptor ligands reduced IGF-1 levels in acromegaly patients (77). Interestingly, GH and IGF-1 deficiencies have been associated with a lower incidence of malignancy (78). Additionally, mutations to enzymes involved in GH/IGF-1 signaling pathways have been linked to increased carcinogenesis (67), although there are dissenting opinions. For example, Gullu et al. (79) argued that the development of TC in acromegaly patients is more closely related to elevated initial GH levels rather than IGF-1 levels, while Zhao et al. (72) found a high prevalence of a BRAF mutation in PTC patients with acromegaly, suggesting the potential pathogenesis of this subgroup. An cohort study conducted in Italy (80) indicated that the risk of DTC was not correlated to GH/IGF-1 levels, but might be associated with BRAF mutations and overexpression of the aryl hydrocarbon receptor. However, Aydin et al. (81) challenged the notion that the BRAF^V600E^ mutation is a causative factor of DTC among acromegaly patients, citing a relatively lower prevalence of this mutation.

### Sex difference and metabolic environment in TC patients with acromegaly

Females generally are at a greater risk of TC than males. However, this association has not been verified in females with acromegaly. A study conducted in Korea (82) reported that TC was the most common malignancy of patients with acromegaly and females were at a greater risk of malignancy, consistent with the prevalence in the overall cohort. However, a similar study (79) reported that the prevalence of TC was higher in males. In most studies, the difference in the incidence of TC between the males and females was not significant. Additionally, in acromegaly patients with controlled disease, GH levels were higher in postmenopausal females than males, while IGF-1 levels were comparable (83).

Xiao et al. (69) found that patients with acromegaly have an increased risk of cancer and acromegaly was associated with diabetes mellitus. GH plays a significant role in glycometabolism and exerts an insulin-sensitizing effect that surpasses the effects of IGF-1 (84). Consequently, long-term exposure to high levels of GH and IGF-1 may lead to insulin resistance and lipodystrophy (85).

In summary, the inconsistent findings of these studies suggest that the predisposition of patients with acromegaly to TC is influenced by factors other than sex hormones. Nonetheless, elevated levels of GH and IGF-1, which are significant hormonal features of acromegaly, may have distinct roles in the development of TC in this population.

### IGF signaling in TC

IGF-1/2 and related receptors have been implicated in the pathogenesis of TC. Studies conducted as early as 30 years ago confirmed that IGF-1 promotes growth of human FTC cells (21). More recent data consistently show significant upregulation of IGF-1 and downregulation of insulin-like growth factor-2 (IGF-2) in TC as compared to normal thyroid tissues (86). Similarly, PTC, but not multinodular nontoxic goiter, is associated with elevated concentrations of circulating IGF-1. Furthermore, IGF-1 receptor (IGF-1R) levels are relatively upregulated in PTC and anaplastic thyroid cancer (ATC) (87). In addition, IGF-1 concentrations were positively associated with the risk of DTC (88). Pidchenko et al. (89) demonstrated that elevated levels of IGF-1 and IGF-2 were correlated with increased insulin production in PTC patients. Furthermore, IGF-1, IGF binding protein-3 (IGF-BP3), and adiponectin levels were correlated to various histologic types of TC. For example, IGF-1 and IGF-BP3 levels were upregulated in patients with intrathyroid invasion and associated with the invasive capacity of TC, while IGF-1, IGF-BP3, and adiponectin levels with type 2 diabetes were correlated with tumor size (90). Interestingly, IGF-I and IGF-IR expression was observed in children and adolescents, and malignant features were correlated with IGF-1R (91).

IGF-1 promotes progression of PTC through the signal transducer and activator of transcription 3 (STAT3) signaling pathway (92). A study conducted by Lv et al. (93) to clarify the mechanisms underlying the onset and proliferation of TC demonstrated that release of IGF-1 by M2-like tumor-associated macrophages promoted metastasis and increased the stemness of ATC cells via insulin receptor-A/IGF1R-mediated activation of the phosphoinositide 3-kinase (PI3K)/alpha serine/threonine-protein kinase (AKT)/mammalian target of rapamycin (mTOR) signaling pathway (93). Furthermore, the long non-coding RNA Linc00210 was reported to enhance the malignant potential of TC cells via modulation of the miR-195-5p/IGF1R/Akt axis (94). Additionally, the disrupted in renal carcinoma 3 was shown to alter progression of DTC by regulating IGF signaling (95).

Despite a compelling preclinical rationale for targeting IGFs in TC, the findings of clinical studies thus far have been underwhelming, which could be attributed to the interplay between IGF signaling and other pathways, leading to resistance against targeted agents designed to inhibit specific components of these intricate signaling networks (96).

## Overlapping activities and interconnectedness of hormone systems

Hormones not only regulate growth of TC cells through shared pathways, but also interact to modulate regulatory effects. Hormone levels differ between TC patients and healthy controls, as some TC patients exhibit abnormal thyroid function, imbalance of sex hormone levels, and endocrine-related diseases, such as diabetes and acromegaly. As mentioned earlier, the synthesis and secretion of these important hormones are regulated by the hypothalamic-pituitary axis. The unique hormone environment of TC cells directly or indirectly affects hormone secretion by the hypothalamus and pituitary gland. However, single hormone monitoring and management is insufficient to control the occurrence and development of TC.

**Figure 2:**
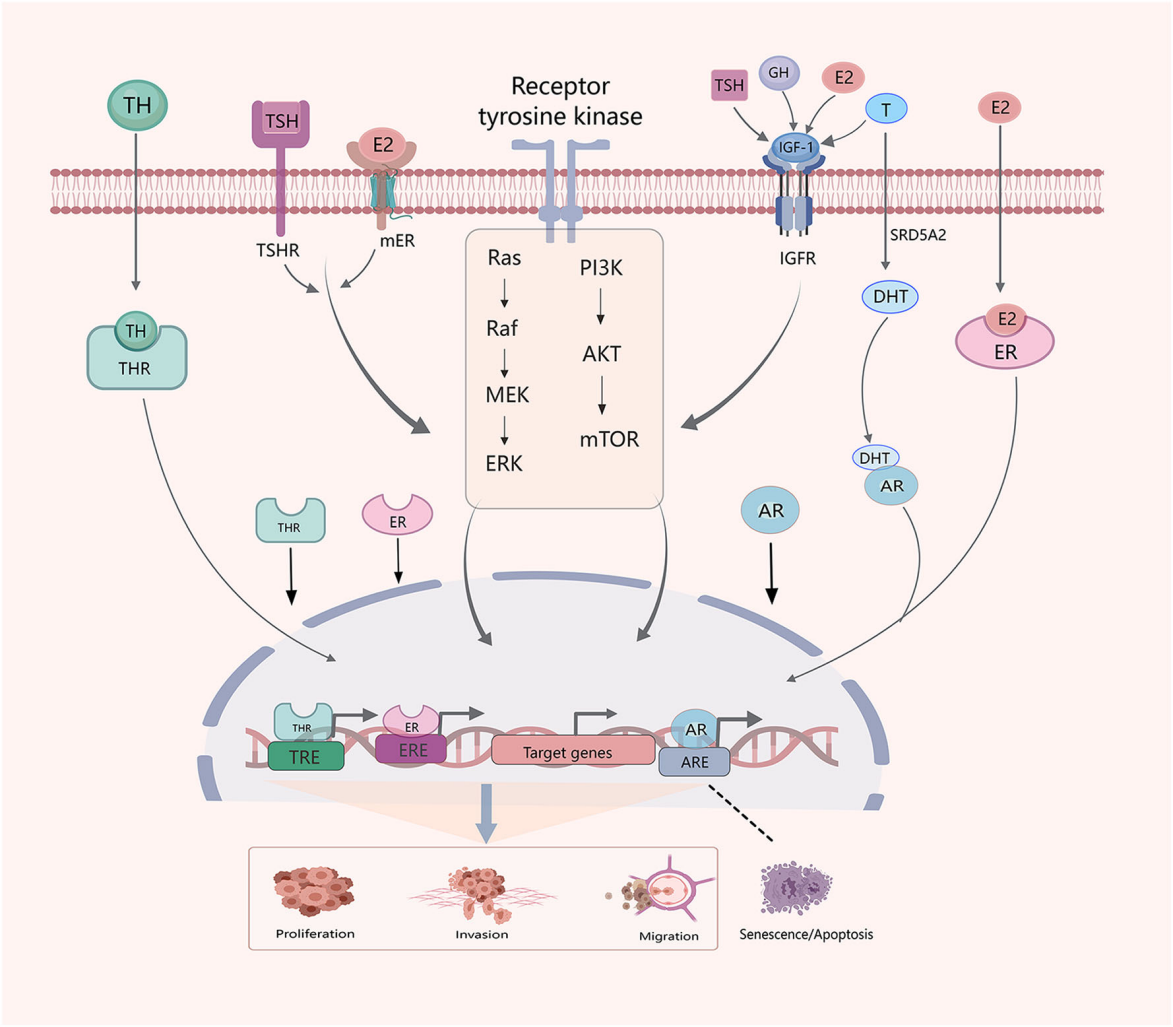
Crosstalk between hormones and hormone systems in the development of TC. Sex hormones, THs, TSH, GH and IGF-1 interact. E2, IGF-1 and TSH influence thyroid development through PI3K/AKT/mTOM and MAPK signaling pathways. Similarly, E2,THs and testosterone (T) regulate progression of TC through classical genomic factors. The SRD5A2 enzyme reduces T to dihydrotestosterone (DHT).

In TC cells, sex steroids, GH/IGF-1, and TSH/TH activate the PI3K/AKT and mitogen-activated protein kinase (MAPK) pathways (**Figure 2**). Recent studies have extensively explored the effects of THs, TSH, and sex hormones of TC, but relatively few studies have investigated the impact and interactions of cortisol, GH, and other hormones in TC.

### GH/IGF axis and sex hormone/receptors

Centrally, sex hormones regulate pituitary secretion of GH and, peripherally, modulate related signaling pathways (97). Estrogens, for example, can reduce IGF-1 levels by inhibiting GH secretion by hepatocytes, but increase central GH secretion (98). Meanwhile, estrogens suppress GH receptor signaling via inhibition of cytokine receptor signaling (98). However, compounds with estrogen-like properties can inhibit some activities of GH while promoting others. Androgens, on the other hand, affect both GH secretion and activities, and also play a role in stimulating the production of IGF-1 (98). The biological function of estrogens, especially E2, are mainly exerted by interactions with ERs. This process is subject to feedback regulation by GH and IGF-1 (99). In men, testosterone concentrations are positively correlated with the regularity of GH secretion, although both testosterone and GH concentrations tend to decrease with increasing age (100, 101).

The close relationship between nutrient metabolism and IGFs expression was demonstrated as early as 1994 (102). Secretion of GH under fasting conditions has sex-specific ramifications (101, 103). A pediatric study conducted by Cicognani et al. (104) found that sex hormones influence IGF-1 levels, which appears to be mediated through GH secretion. A study by Papatheodorou et al. (105) revealed an association between circulating levels of E2, sex hormone-binding globulin (SHBG), and IGF-BP3 in males in the United States. In addition, both IGF-1and ER mediate various biological processes via the PI3K signaling pathway that are crucial for the onset and progression of TC. Additionally, metformin may be useful for treatment of differentiated or poorly differentiated TC, which may involve regulation through the ER or AR signaling pathway, as demonstrated in prostate cancer (106).

The effects of complicated hormone levels on metabolism, particularly glycoproteins and lipids, have been implicated in progression of TC. Both IGFs and estrogen have been found to influence glycolysis in TC. For example, Huang et al. (107) suggested that the fat mass and obesity-associated protein inhibits expression of apolipoprotein E through insulin-like growth factor binding protein 2-mediated m6A modification, which may inhibit glycolytic metabolism in PTC by modulating the interluekin-6/Janus kinase 2/STAT3 signaling pathway, consequently suppressing tumor growth (107). Similarly, Zhu et al. (108) demonstrated that estrogen increases malignant activities as well as glycolysis in PTC cells by inhibiting expression of FAM111 trypsin-like peptidase B (FAM111B) and proposed that the E2/DNA (cytosine-5)-methyltransferase 3B/FAM111B axis is crucial for regulating the progression of PTC (108). Collectively, these findings suggest crosstalk between sex hormones and the GH/IGF axis in TC, particularly PTC.

### TSH/THs and sex hormone/receptors

Extranuclear signaling pathways mediated by THs, estrogens, and androgens regulate various biological processes (109). In certain types of tumors, THs and sex steroids exhibit interacting and overlapping effects. For instance, THs have been shown to influence concentrations of SHBG, as evidenced by elevated testosterone and SHBG levels in males with hyperthyroidism (110). Similarly, the estrogen inhibitor tamoxifen was found to lower serum T3 levels in adult female rats. Moreover, sex-specific relationships between TSH and TH levels were observed in children and adolescents (111). Specifically, this study reported that mean logTSH and freeT3 levels were significantly higher in males than females, while age was negatively correlated with thyroid function in both males and females, and with freeT4 levels in males (111). Currently, there is no evidence linking sex hormones to differences in TSH and TH levels between males and females.

In cancer cells, THs and estrogen signaling display significant crosstalk through promoter cross-reactivity (112). Similarly, THs interact with the AR promoter region and affect the responsiveness of androgen by increasing AR expression. Thyroid response elements have been identified in the promoter regions of AR and several androgen-related genes (113). Estrogens, especially E2, and THs have overlapped biologic effects in TC, although the underlying mechanisms remain unclear. Some reports have suggested that estrogen-related cancers may be permissively modulated by TH and ER, especially ER-positive TC (27). Administration of estrogens may negatively influence treatment of relapsed DTC in both males and females. Estrogens and THs demonstrated an additive effect on tumor growth and development in postmenopausal females with recurrent differentiated tumors after estrogen replacement and TSH suppression (27). Meanwhile, E2 was shown to significantly inhibit TSH-induced differentiation of progenitor cells and expression of the sodium/iodide symporter (46). Furthermore, E2 obviously decreased levels of thyroid differentiation markers, including TSHR, indicating impaired thyroid differentiation (46).

Estrogens and TSH/TH exhibit significant effects on BRAF and p53 activities in TC tumor cells. The BRAF^V600E^ mutation was significantly more common in tumors expressing TSH (114) and was shown to override BRAF-induced senescence, thereby promoting tumor progression via downregulation of p53 expression in PTC (115). Additionally, cell lines carrying the BRAF^V600E^ mutation demonstrated increased metastatic potential in response to E2 (48). In the BRAF^V600E^ group, the ERα/ERβ ratio was elevated in younger participants (≤50 years) (48). Furthermore, E2 was found to synergistically activate the tyrosine kinase pathway in TC cells with the RET fusion and BRAF mutation (37). Moreover, thyroid transcription factor-1, ER, PR, and p53 were co-expressed in most young females (15–34 years) with PTC (116).

Additionally, sex hormones and TSH regulate the development of TC via the MAPK signaling pathway. Estrogen activates MAPK-dependent serine phosphorylation of nuclear ERα. Similarly, a study by Jiang et al. (51) showed that testosterone promotes malignancy via the MAPK (p38/JNK) signaling pathway. TH is a MAPK-dependent growth factor with anti-apoptotic effects. When THs are activated, the MAPK (ERK1/2) signaling pathway induces serine phosphorylation of several nucleoproteins, including nuclear ERβ (117). An *in vitro* study reported that microRNA 106a, which regulates expression of the TSH receptor, is associated with the proliferation, apoptosis, differentiation, and iodine uptake of TC cells by modulating the MAPK signaling pathway (118). Furthermore, ERs have been shown to modulate the PI3K/AKT/mTOR pathway, thereby influencing proliferation of TC (41). Similarly, activation of TSH-TSHR signaling has been found to play a role in increasing mobility and dedifferentiation of TC cells via crosstalk with the PI3K/AKT/mTOR signaling pathway (119).

### IGF1 and TSH/TH

Similar to TSH, IGF-1 is also considered a risk factor in TC (120). An *in vitro* study (96) demonstrated that IGF-1R enhances TSH-induced activation of thyroid-specific genes, particularly the sodium/iodide symporter. This process is facilitated by the ERK1/2 or AKT pathways. Additionally, TSH interacts with IGF-1 (22). The co-activities of TSH and IGF-1 promote growth of human FTC cells (21). Moreover, TSHR and IGF-1R have been observed to co-immunoprecipitate in both orbital and thyroid tissues, indicating formation of a functional complex (121). Furthermore, TSH and insulin/IGF-I act synergistically to increase proliferation and growth of thyroid cells, which are primarily mediated through the cyclic adenosine monophosphate, PI3K, and MAPK pathways (122, 123).

## Crosstalk of hormone systems as a risk factor for TC

**Figure 3:**
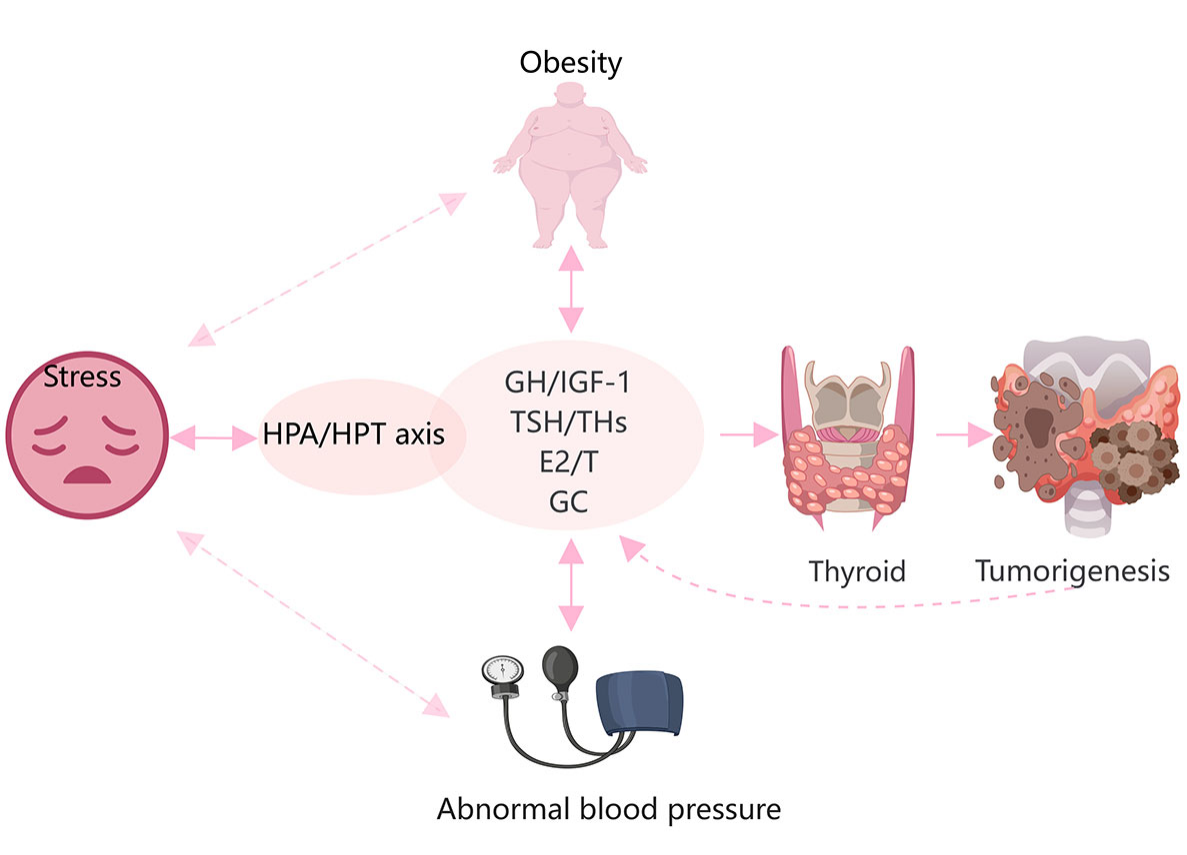
Hypertension, obesity, and stress are closely related to hormonal changes, and promote the onset and progression of TC. Glucocorticoids(GC), THs, sex steroids, and the GH/IGF-1 axis are directly and indirectly involved in the development of obesity and hypertension. Stress is mainly regulated by the HPA and HPT axes. These factors are involved in the occurrence and development of TC.

Increasing evidence suggests that obesity, stress, and high blood pressure increase the risk of TC (10, 124). Various hormones are also activated in response to these stressors (**Figure 3**). Obesity is a complex physiological condition that is regulated by various hormones, but can also lead to hormonal imbalances and homeostatic disruptions. In general, obesity, central fat distribution, stress, depression, and unstable blood pressure are closely related and may contribute to several pathological effects, such as hyperinsulinemia, increased aromatase activity, chronic inflammation, altered immune responses, and oxidative stress (125). IGF-1, sex hormones, TSH, and cortisol directly contribute to these processes and play significant roles in thyroid tumorigenesis.

Obesity is an established risk factor for DTC in females but not males (126). Both obesity and TC are more frequent in females than males worldwide, implying the involvement of estrogens (127, 128). In fact, serum levels of ERα are increased in obese females (128). However, various factors other than sex, such ethnicity and age, especially adolescence, may influence the association between obesity and the risk of TC (125). An imbalance of estrogens and androgens may be responsible for the greater risk of TC in obese individuals (125). As a well-known risk factor for diabetes, obesity is characterized by insulin resistance and hyperinsulinemia (128). IGF-1 and IGF-2 mediate the functions of insulin as well as the obesity process (129). Marcello et al. (126) reported that higher consumption of animal proteins and carbohydrates contribute to obesity and a greater risk of DTC via a mechanism possibly related to upregulation of IGF-1.

Furthermore, the HPA and HPT axes are also involved in the regulation of obesity, stress, and depression (130, 131). Accumulating epidemiological data demonstrate an independent association between TSH levels and obesity (125). Additionally, there is a parallel increase in the incidence of TC among both adults and children in conjunction with the rising levels of stress in contemporary society. Prolonged secretion of glucocorticoids due to stress can potentially induce chronic inflammation and suppress immune responses. Stress can lead to dysregulation of the thyroid gland through crosstalk between the HPA and HPT axes. However, further investigations are needed to determine whether this association is involved in the onset and progression of TC (132).

### Hormone regulation in TC at different ages

Hormone levels change with age. Typically, children and adolescents experience hormone surges during periods of rapid growth and development. Adult and middle-aged women usually have relatively stable hormone levels until perimenopause or menopause, when hormone levels once again fluctuate. Hormonal changes may explain why TC cells in adults also exhibit milder characteristics, with tumors less aggressive and less able to migrate than in children and the elderly (**Table 3**).

**Table 3:** Characteristics of the hormonal environment and TC in different age groups.

	Children and adolescents	Adults	Elderly
Hormonal environmental characteristics	1, More sensitive to hormones 2, Have better reactivity to hormones 3, Hormone secretion significantly fluctuates	1,Hormone levels are relatively stable 2,Hormone secretion is regular	Decreased hormone reactivity is often accompanied by decreased secretion
GH secretion	Higher GH level	GH levels decrease with age	
Sex hormones secretion	Sex hormone levels are significantly increased during puberty	Sex hormone levels reach highest levels	Sex hormone levels are decreased
Characteristics of TC	1, Higher lymph node metastasis rate 2, 95% of pathological types are PTC 3, Favorable long-term prognosis	1, Prognosis is good 2, Tumor invasiveness and migration ability are relatively poor	1, Prognosis is relatively poor 2, Increased rate of undifferentiated TC 3, Distant metastasis is more common

### Hormone regulation in TC among younger populations

DTC is the most frequent malignant tumor of the endocrine system in children and adolescents. TC, especially DTC, is typically more aggressive in pediatric patients than adults. Nevertheless, the prognosis of pediatric TC patients is often positive, even for those with advanced disease. However, the prognosis of persistent or recurrent disease is often poor (133). Therefore, it is crucial to elucidate the pathogenesis of TC in young individuals and effectively manage associated risk factors to optimize treatment.

At puberty, the HPG axis interacts with the GH/IGF-1 axis to facilitate physical growth spurts (134). In addition, GH and insulinomimetic signals are regulated by sex steroids. Androgens, for example, induce IGF-1 activation in bone, muscle, and skin tissues, while suppressing IGF-1R expression in adipose tissues. Thus, regulation of GH and IGF-1 is determined by the specific type of sex hormone and target tissue. In contrast, GH and insulin upregulate AR and ER in an organ-specific manner. Consequently, interactions of sex steroids with the GH/IGF axis can increase susceptibly to TC in children and teenagers.

Sex steroids, rather than TSH, play more significant roles in thyroid development in children and adolescents (16). Notably, during puberty in females, characteristic changes to glycosylation are associated with sex hormones, particularly E2. These modifications may create a favorable TME for TC (49). A case-control study conducted by Kim et al. (135) revealed a relationship between obesity and TC in a young Korean population (18 years old). Besides obesity, other risk factors for TC include adolescence (125). A case-control study by Suzuki et al. (136) demonstrated a positive association between body weight and an increased risk of TC in children and adolescents, particularity in males. However, post puberty, the risks of TC and persistent disease decrease. The post-puberty period is strongly associated with the clinical behavior of TC and males are at a greater risk for persistent disease (137). High levels of TSH are generally considered a risk factor for TC. However, this regulatory mechanism appears to be more prevalent in adults than teenagers. Suzuki et al. (111) reported that TSH levels were associated with age and thyroid function in children and adolescents with pediatric thyroid carcinoma, and age was negatively correlated with thyroid function in both male and female teenagers. Moreover, lower logTSH levels and higher antithyroglobulin levels were independent risk factors for the development of thyroid nodules and age was positively correlated with free T4 levels in young males (111). These findings suggest that teenage males may be more susceptible to TC than adult males.

ERα expression is lower in adolescents with TC, while hormone receptor levels in females are not related to sex, American Thyroid Association risk score, persistent structural disease, or pubertal status (137). Therefore, female hormones and receptors may not be major factors in the progression of TC at puberty.

Additionally, IGF-1 and IGF-1R are associated with the development of TC in younger populations, as high expression of IGF-1R enhances the aggressiveness of cancer cells (91). Circulating levels of GH, IGF-1, sex hormones, and THs may directly or indirectly affect the expression levels of IGF-1 and IGF-1R in tumor tissues.TC tends to be more aggressive in children and adolescents with higher GH and IGF-1 levels. Moreover, the unique psychological changes and stress experienced during adolescence may amplify the interplay between hormonal systems, particularly the HPA and HPT axes. Hence, further studies are warranted to investigate the characteristics of TC and associated risk factors in children and adolescents.

### Hormone regulation in TC among the elderly

According to the American Joint Committee on Cancer, age at diagnosis is associated with staging of DTC and advanced age is associated with compromised prognosis. The pathogenesis of DTC is complex in older patients and not well understood. Thus, further studies are needed to explore the roles of hormone systems in the elderly.

Younger people usually have substantial physiological reserves, which are gradually depleted with aging because of reduced hormone requirements. During senescence, hypopituitarism may be a physiological process. Older patients are more susceptible to growth hormone deficiency, gonadotropin deficiency, and hypothyroidism (138). Pituitary hormone deficiency increases with age. DTC cells are regulated by multiple hormones. Older people experience a relatively nutrient-poor environment due to lower hormone levels and less physiological reserves, which can increase the susceptibility of DTC cells to stress, thereby enhancing invasion and migration.

The prevalence of TC is increased in patients with hypothyroidism (139). Subclinical hypothyroidism was identified as an independent risk factor for extrathyroidal extension in patients with PTC. The risk of extrathyroidal extension associated with subclinical hypothyroidism is reportedly higher in males than females (140). More importantly,hypothyroidism is the most frequent endocrine disease in the elderly, with an increased prevalence in women as compared to men (141, 142). The most frequent cause of hypothyroidism in the elderly is autoimmune thyroiditis (143). Older men have higher baseline TSH levels and lower T3 and free T4 serum levels than younger men. Older people also have lower TSH responses to thyrotropin-releasing hormone. Furthermore, the inhibitory effect of glucocorticoids on TSH secretion is reduced with aging in men (144). This evidence suggests that the elderly, especially older men, may exhibit poorer hormonal responsiveness. The secretion of hypothalamic-pituitary axis and target organ hormones, especially glucocorticoids and TSH, may be chaotic. In this hormone environment, TC cells are more stressful, and the cancer-promoting effects of hormones may be more obvious.

Undifferentiated TC is more common in the elderly. TSH and IGF-1 are major regulators of thyroid cell differentiation in adults (19). IGF-1 is crucial for differentiation of murine embryonic stem cells to thyrocytes (145). The lower level of IGF-1 in the elderly may increase the dedifferentiation of tumor cells in TC, which may explain why 95% of TCs are well differentiated in children and adolescents. For example, high total IGF-2 levels accompanied by low serum levels of IGF-I and GH have been associated with poorly differentiated TC in the elderly (146). In addition, postmenopausal women have increased ERα expression (147), which may be involved in more aggressive behavior. Similarly, decreased estrogen levels and increased levels of follicle-stimulating hormone are associated with epidermal growth factor receptor expression and activation in postmenopausal women with DTC (148). This status is involved in the recurrence of PTC (148). In summary, differences in the hormone environment, including fluctuations in thyroid function, suppression of the GH/IGF-1axis, and changes to estrogen and ER levels, partially explain why TC is more common and aggressive in older people.

## Conclusion and future perspectives

The thyroid is a complex endocrine gland that produces a variety of hormones. The onset and progression of TC, particularly DTC, are regulated by various hormone systems. The interactions of various hormones influence thyroid tumorigenesis. In pediatric patients, the development of TC, particularly DTC, is dependent on the interactions of sex hormones and GH/IGF-1, especially during puberty due to specific physiological needs. Thus, further investigations with the use of *in vitro* and animal models should focus on the mechanisms underlying the crosstalk between various hormones and hormone systems in the onset and progression of TC.

## Author contributions

L-HC: Writing – original draft, Writing – review & editing. TX: Writing – original draft. QL: Writing – original draft. Y-RG: Writing – original draft. C-ZS: Writing – review & editing.

## Glossary

**Table:**

ARE	Androgen response element
ARs	Androgen receptors
AKT	Alpha serine/threonine-protein kinase
ATC	Anaplastic thyroid cancer
CPAs	Cortisol-producing adenomas
CRH	Corticotropin-releasing hormone
DHT	Dihydrotestosterone
DTC	Differentiated thyroid carcinoma
ER	Estrogen receptor
ERα	Estrogen receptor alpha
ERβ	Estrogen receptor Beta
ERE	Estrogen response element
E2	Estradiol
EMT	Epithelial-mesenchymal transition
ERK1/2	Extracellular signal-regulated kinases 1 and 2
FAM111B	FAM111 trypsin-like peptidase B
FTC	Follicular thyroid cancer
GH	Growth hormone
GC	Glucocorticoid
GR	Glucocorticoid receptor
GHRH	Growth hormone-releasing hormone
HPA	Hypothalamic-pituitary-adrenal
HPG	Hypothalamic-pituitary-gonadal
HPT	Hypothalamic-pituitary-thyroidal
IGF-1	Insulin-like growth factor-1
IGF-2	insulin-like growth factor-2
IGF-1R	Insulin-like growth factor-1 receptor
IGF-BP3	Insulin-like growth factor binding protein-3
mTOR	Mammalian target of rapamycin
MAPK	Mitogen-activated protein kinase
NADPH	2,4-dienoyl CoA reductase 1
NF-kB	The nuclear factor kB
PD-L1	Programmed death-ligand 1
PR	Progesterone receptor
PTC	Papillary thyroid carcinoma
PI3K	Phosphoinositide 3-kinase
ROS	Reactive oxygen species
STAT3	Signal transducer and activator of transcription 3
SHBG	Sex hormone-binding globulin
T	Testosterone
TC	Thyroid cancer
TSH	Thyroid stimulating hormone
THs	Thyroid hormones
TSHR	Thyrotropin receptor
T3	Triiodothyronine
T4	Thyroxine
TME	Tumor microenvironment.
TRE	Thyroid hormone response element
